# Posttraumatic growth in children aged 8–18 years with malignancies in China

**DOI:** 10.1186/s12887-022-03799-w

**Published:** 2022-12-29

**Authors:** Yi-Xuan Liu, Qian Liu, Lu Yu, Lin Mo

**Affiliations:** 1 Department of Nursing of Children’s Hospital of Chongqing Medical University, National Clinical Research Center for Child Health and Disorder, Ministry of Education Key Laboratory of Child Development and Disorders, Chongqing Key Laboratory of Pediatrics, Chongqing, 400014 China; 2 Department of Outpatient, Children’s Hospital of Chongqing Medical University, ChongQing, China

**Keywords:** “Posttraumatic growth”, “Nomogram”, “Prediction model”, “Children”, “Malignancies”

## Abstract

**Objective:**

To establish a nomogram prediction model for posttraumatic growth (PTG) in children aged 8–18 years with malignancies in China and to convenient intuitively judge psychological tendencies.

**Methods:**

We recruited 358 children aged 8–18 years with malignancies in China as the study participants. Data from 250 cases collected from June 2019 to November 2019 were used as the model group, data from 108 cases collected from December 2019 to January 2020 were used as the validation group. Logistic regression was used to analyze the influencing factors of PTG in the model group. A prediction model was then established using a nomogram. The centrality measurement index(C-index) and receiver operating characteristic curves (ROC) were used to verify the model.

**Results:**

Among the 250 children in the model group, 65 children with malignancies had PTG, with an occurrence of 26%. The model showed that the child’s age, diagnosis, coping style and self-efficacy level and the educational level of the caregiver were core predictors of PTG (*P* < 0.05). The ROC of the model was 0.837, the best cutoff value was 0.566. The C-indexes of the internal and external validation were 0.837 (95% CI: 0786 ~ 0.886) and 0.813 (95% CI: 0732 ~ 0.894), respectively.

**Conclusions:**

The prediction model of PTG in children aged 8–18 years with malignancies in China has good discrimination and consistency and can accurately predict PTG. It can be used to clinically assess the psychological status of children in the future.

## Introduction

In recent years, the number of new cases of children with malignancies worldwide has been approximately 168,000 per year, and the annual incidence rate is increasing at a rate of 0.7% [1] Yet, the 5-year survival rate for children with malignancies is as high as 85% [2]. The growing number of survivors represents children and their families facing a range of problems, such as secondary tumors, late complications [3], the development of poor behavior [4] and even death. Moreover, according to the relevant literature, children possess a definite cultural knowledge base after the age of 8 years to have an initial understanding of society and personal thoughts [5]. There is also the ability to self-report the occurrence of events [6]. However, during this time, children are at a specific stage of transition to adulthood and more likely to feel stress when faced with events or experiences in their growing environment. Cancer issues can have a serious impact on their future health-related quality of life [7, 8]. Research has shown that children with cancer show both negative and positive psychological responses in their fight against cancer. Negative psychological changes include anxiety [9], depression [10] and suicidal ideation [11], and one positive psychological change is posttraumatic growth (PTG). PTG a collection of positive psychological and spiritual changes experienced by individuals during the follow-up process after a traumatic event or situation [12]. However, previous studies have focused on negative psychological changes in children, ignoring the positive reflections during their cancer experience that are beneficial to their development [13, 14]. Therefore, it is of great practical importance to clarify the factors affecting PTG in children aged 8–18 years with malignancies in China in order to inform guide clinical caregivers in implementing interventions.

At present, several studies [15, 16] have shown that PTG is a common phenomenon for children with malignancies and their families. The positive reflections and beliefs brought about by PTG could help survivors successfully resume their daily lives in the follow-up survival process [17]. One study [18] also demonstrated that PTG can be an important predictor of quality of life in children with malignancies, but further exploration is needed [19]. Furthermore, due to the different cultural backgrounds in different countries, there is disagreement as to whether factors such as religion and gender affect PTG [20]. Many studies of PTG in children with malignancies have focused on caregiver and disease factors [21, 22]. Our pre-exploration study also identified age, region, self-efficacy, social support, and coping styles as influential factors in PTG [23], but the sample size was too small (64 cases) to provide high-quality evidence. It is evident that the independent influences on PTG are not well defined in domestic and international studies. Moreover, longitudinal studies are still relatively lacking, and there is wide variation in the choice of longitudinal time points and methods of data analysis, leaving the pattern of PTG development in cancer patients poorly understood. In addition, the existing psychological interventions are mainly aimed at children whose problems have already occurred, making it difficult to provide high-quality evidence for the development of an early intervention program for PTG. This research aims to clarify the independent predictors of PTG in children with malignancies in China and construct a measurable predictive tool to address the above issues.

In summary, it is important to be able to predict the occurrence of PTG for the quality of life and mental health of children with malignancies in China. We screened the core influences of PTG to develop a nomogram prediction model to predict PTG. This study will allow clinical caregivers to more clearly assess the probability of PTG, which can be helpful for children to establish good PTG and quickly return to ordinary life.

## Materials and methods

### Study participants

Starting from June 2019 to January 2020, children with malignancies from the inpatient unit of the Department of Hematology and Oncology Surgery at a tertiary care children’s hospital were recruited for an online survey. The inclusion criteria were as follows: (1) patients aged 8–18 years (this age group has self-reporting capabilities) [6]; (2) patients diagnosed with cancer by the standard guidelines for the diagnosis and treatment of childhood malignancies [24]; and (3) children who provided informed consent. The exclusion criteria were (1) children suffering from severe organic or cognitive impairment and (2) children who were unable to understand the questionnaire. The final recruited children will be divided into two groups: the modeling group and the validation group. The model group was used to construct the prediction model; the verification group was used to evaluate the applicability of the model. This study was approved by the Institutional Review Board of Children’s Hospital of Chongqing Medical University, China [2019 (year) No. 8]. All the study subjects and caregivers had the right to withdraw at any time during the study.

### Data collection

Prior to the investigation, the researchers introduced themselves to the children and their caregivers and informed them of the purpose and content of the study. In addition, data adhered to the principle of confidentiality, private information such as the child’s name was not disclosed, the primary caregiver and children signed informed consent form, and the survey was completed anonymously. Each study participant could only submit one questionnaire, which prevented the possibility of double registration. To ensure the quality of the participants’ responses, we required that each question be answered. Variables that were not understood were explained in detail to guide the participants to choose the options that fit their situation.

To better screen the core predictors of PTG, we selected the following basic information potentially relevant to PTG as independent variables based on previous studies and ecosystem theory, including the child’s age, gender, diagnosis, time of hospitalization, stage of disease, area of residence, temperament, whether or not the religious, primary caregiver, education level of the caregiver, family economic status, family parenting style, and type of family. Among them, family parenting style, family type and temperament of the child were judged mainly in relation to the actual situation of the child and family for subjective selection. Some of the above variables are not core predictor variables of PTG, therefore, only preliminary determination of the variables was made in this study. The typology of specific variables can be seen in Table 1.

**Table 1:** Univariate analysis of the PTG of children with malignancies (*N* = 250)

Variable	No PTG(*N* = 185)		PTG (*N* = 65)		χ^2^	P
	n	%	n	%		
Gender						
Boys	106	57.29	37	56.92	0.003	0.958
Girls	79	42.70	28	43.07		
Stage of disease						
Diagnosis period	36	19.46	15	23.07	8.983	0.011*
Treatment period	127	68.64	33	50.76		
Rehabilitation period	22	11.89	17	26.15		
Area of residence						
Rural	118	63.78	51	78.46	4.731	0.030*
Urban	67	36.21	14	21.53		
Caregiver						
Parent	172	92.97	64	98.46	1.801	0.180
Not parent	13	7.02	1	1.53		
Educational level of the caregiver						
Primary school	62	33.51	14	21.53	15.448	<0.001*
Junior and senior high school	113	61.08	37	56.92		
Bachelor’s degree or higher	10	5.40	14	21.53		
Diagnosis						
Nonsolid tumor	102	55.13	51	78.46	11.022	0.001*
Solid tumor	83	44.86	14	21.53		
Religiosity						
No	174	94.05	58	89.23	1.031	0.310
Yes	11	5.94	7	10.76		
Family parenting style						
Doting	60	32.43	8	12.30	9.905	0.007*
Autocratic	17	9.18	7	10.76		
Authoritative	108	58.37	50	76.92		
Family type						
Stem family	100	54.05	41	63.07	2.490	0.477
Nuclear family	50	27.02	13	20.00		
Reconstituted family	8	4.32	4	6.15		
Single-parent family	27	14.59	7	10.76		
Family economic status (monthly)						
< 3000	81	43.78	26	40.00	0.281	0.869
3000 ~ 5000	80	43.24	30	46.15		
> 5000	24	12.97	9	13.84		
Temperament of the child						
Extroverted	98	52.97	44	67.69	4.247	0.039*
Introverted	87	47.03	21	32.31		
Coping style of the child						
Positive	73	39.45	44	67.69	15.399	<0.001*
Negative	112	60.54	21	32.30		
Level of self-efficacy						
Low	96	51.89	9	13.84	32.666	<0.001*
Medium	82	44.32	46	70.76		
High	7	3.78	10	15.38		
Level of depression						
Normal	102	55.13	54	83.07	16.429	<0.001*
Mild	41	22.16	7	10.76		
Moderate or above	42	22.70	4	6.15		
Level of anxiety						
Normal	82	44.32	47	72.30	15.234	<0.001*
Mild	18	9.72	4	6.15		
Moderate or above	85	45.94	14	21.53		
Level of stress						
Normal	136	73.51	57	87.69	9.621	0.008*
Mild	19	10.27	7	10.76		
Moderate or above	30	16.21	1	1.53		

*is *P*<0.05

### Measurement tools

PTG was measured by the Posttraumatic Growth Assessment Scale (PTGI), which is a 21-item questionnaire with five factors [25]. The participants responded on a 6-point Likert scale, with 0 for no change and 5 for complete change. The total score ranges from 0 to 105, with higher scores indicating higher levels of PTG. In this study, the total score ≥ 54.23 indicates PTG [26]. The Cronbach’s alpha value of PTG was 0.91 in our sample.

The level of negative emotions was measured by the 21-item Depression Anxiety Stress Scale (DASS-21) [27] with three dimensions: depression, anxiety and stress. Each item is scored from 0 (never) to 3 (almost). A higher score indicates more serious negative emotional symptoms. In this study, the total Cronbach’s alpha value of this scale was 0.88.

The children’s coping styles were calculated by the Simplified Coping Style Questionnaire [28]. The 4-point scale from 0 (never) to 3 (quite a lot) has 20 items. Coping tendency = positive coping standard score (z score) - negative coping standard score (z score). Z score = (dimension individual total score - dimension mean) / dimension standard deviation. If the coping tendency value is greater than 0, it means that the subjects mainly adopt positive coping style in the state of stress. In this study, the Cronbach’s alpha values of positive, negative dimension and total score were 0.81, 0.68 and 0.79 respectively.

The children’s self-efficacy was measured by the General Self-Efficacy Scale (GSES) [29], which includes one dimension and 10 items. Each item is scored from 1 (quite wrong) to 4 (quite right), and the total score ranges from 10 to 40. The standard score is the total score divided by the total number of items. The higher the standard score is, the higher the self-efficacy is. According to the score index, the self-efficacy level was divided into three grades: high (≥80%), medium (60% ~ 80%) and low (< 60%). The score index was calculated as follows: actual standard score/theoretical maximum standard score* 100%. The total Cronbach’s alpha value of the scale in this study was 0.81.

### Statistical analysis

All statistical analysis was performed using R (version 4.2.1) and SPSS 24.0. In single factor analysis, the count data are described as the frequency and percentage (%). The K-S test was used to test the normality of the data. The measurement data are described as the mean ($\overline{\chi}$ ± standard deviation (SD). the Mann–Whitney U rank sum test was used to compare the difference between two samples of nonnormally distributed measurement data. Then, we assigned values to variables with *P* < 0.1 in the univariate analysis and those after clinical consideration. Among them, dummy variables were set for polytomous variables and referenced to the first category. For identifying the final prediction model, a forward-stepwise method was used to select predictors of PTG in multivariable logistic regression. The nomogram and calibration curves were constructed and evaluated using the package with the “Rms” and “Hmisc”. The C-index and ROC curves were used to evaluate the model with a test level of α = 0.05. Internal verification was evaluated using the bootstrapping validation method.

## Results

### Study population

A total of 358 children were eventually completed the survey. Data from 250 cases collected from June 2019 to November 2019 were used as the model group, data from 108 cases collected from December 2019 to January 2020 were used as the validation group. The PTG scale score was 43.42 ± 17.43 for the model group consisting of 250 children, 143 of whom were boys. The mean age of the group without No PTG was (10.97 ± 2.18) and the mean age of the group with PTG was (11.98 ± 2.19), comparing the two groups (t = − 3.200, *p* = 0.001). The mean time of hospitalization in the group without No PTG was (8.00 ± 16.00) and in the group with PTG was (10.00 ± 17.50), comparing the two groups (t = − 0.204, *p* = 0.839). Among them, 41 (16.4%) had lymphoma, 153 (61.2%) had leukemia, 20 (8.0%) had sarcoma, and 36 (14.4%) had other tumors (including teratomas, nasopharyngeal carcinomas, ovarian yolk sac tumors, neuroblastomas, etc.) For statistical convenience, the children were divided into those with solid and nonsolid tumors (Table 1).

### Factors associated with PTG in Chinese children aged 8–18 years with malignancies

Gender was not shown to be statistically associated with PTG in the univariate analysis. However, based on clinical professional judgment, it was considered for inclusion. The final results showed that the most meaningful predictors of PTG were level of self-efficacy, educational level of the caregiver, age, diagnosis and coping style of the child (Table 2).

**Table 2:** Logistic regression analysis of PTG in Chinese children with malignancies

Variable	*β*	*SE*	*Wald*	*P*	*OR*	95% *CI*
Constant	−1.908	1.247	2.341	0.126	0.148	–
Age (boys)^a^	0.209	0.079	6.954	0.008*	1.233	1.055 ~ 1.440
Educational level of the caregiver (Primary)^a^			14.840	0.001*		
Junior or senior high school	0.459	0.398	1.331	0.249	1.582	0.726 ~ 3.448
Bachelor’s degree or higher	2.314	0.610	14.404	0.000*	10.111	3.061 ~ 33.398
Diagnosis (Nonsolid tumor)^a^	−1.281	0.393	10.615	0.001*	0.278	0.129 ~ 0.660
Coping style of the child (Positive)^a^	−1.113	0.364	9.356	0.002*	0.328	0.161 ~ 0.670
Level of Self-efficacy (Low)^a^			21.113	0.000*		
Medium	1.600	0.435	13.538	0.000*	4.954	2.112 ~ 11.618
High	2.935	0.702	17.499	0.000*	18.816	4.757 ~ 74.421

*β* is the regression coefficient; *SE* is the standard error; *95% CI* is the confidence interval; ^a^ is the reference group; ^*^is *P*<0.05

### Prediction model for PTG in Chinese children aged 8-18 years with malignancies

**Fig. 1:**
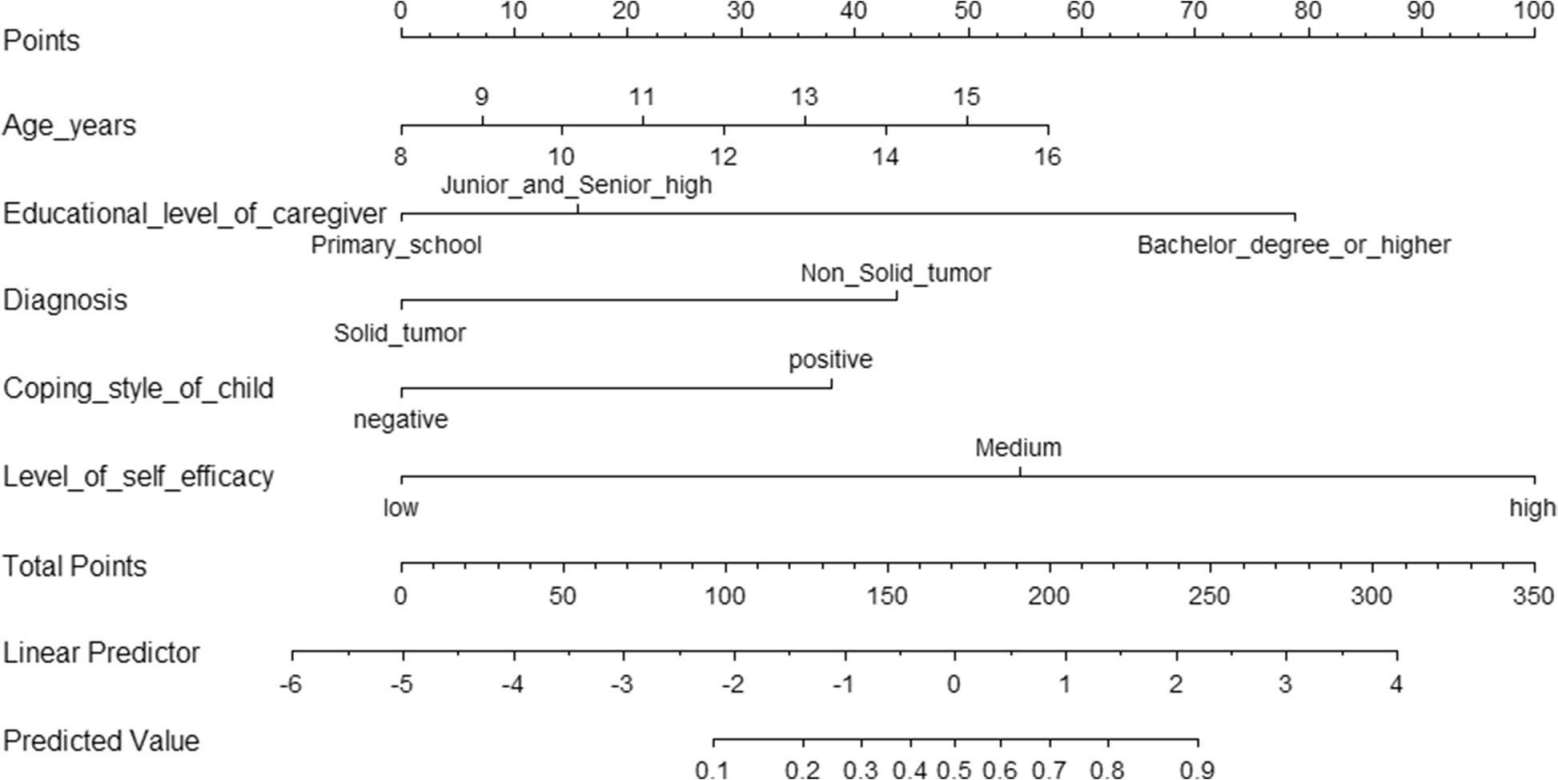
Establishment of a nomogram model for predicting PTG in Chinese children aged 8-18 years with malignancies

**Fig. 2:**
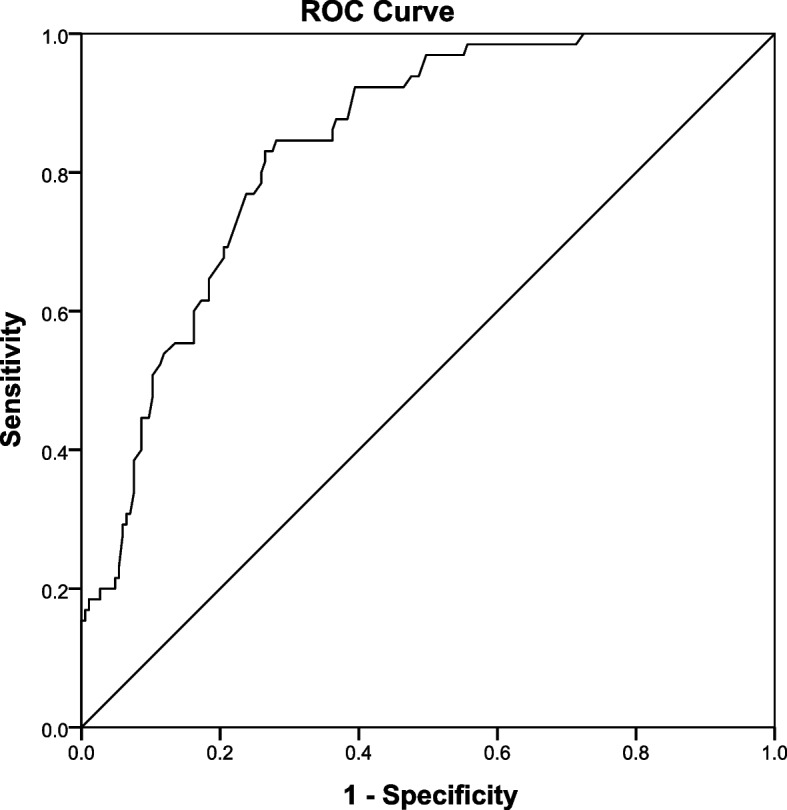
Nomogram model predicted the ROC curve of PTG in children aged 8-18 years with malignancies. AUC, area under the curve

**Fig. 3:**
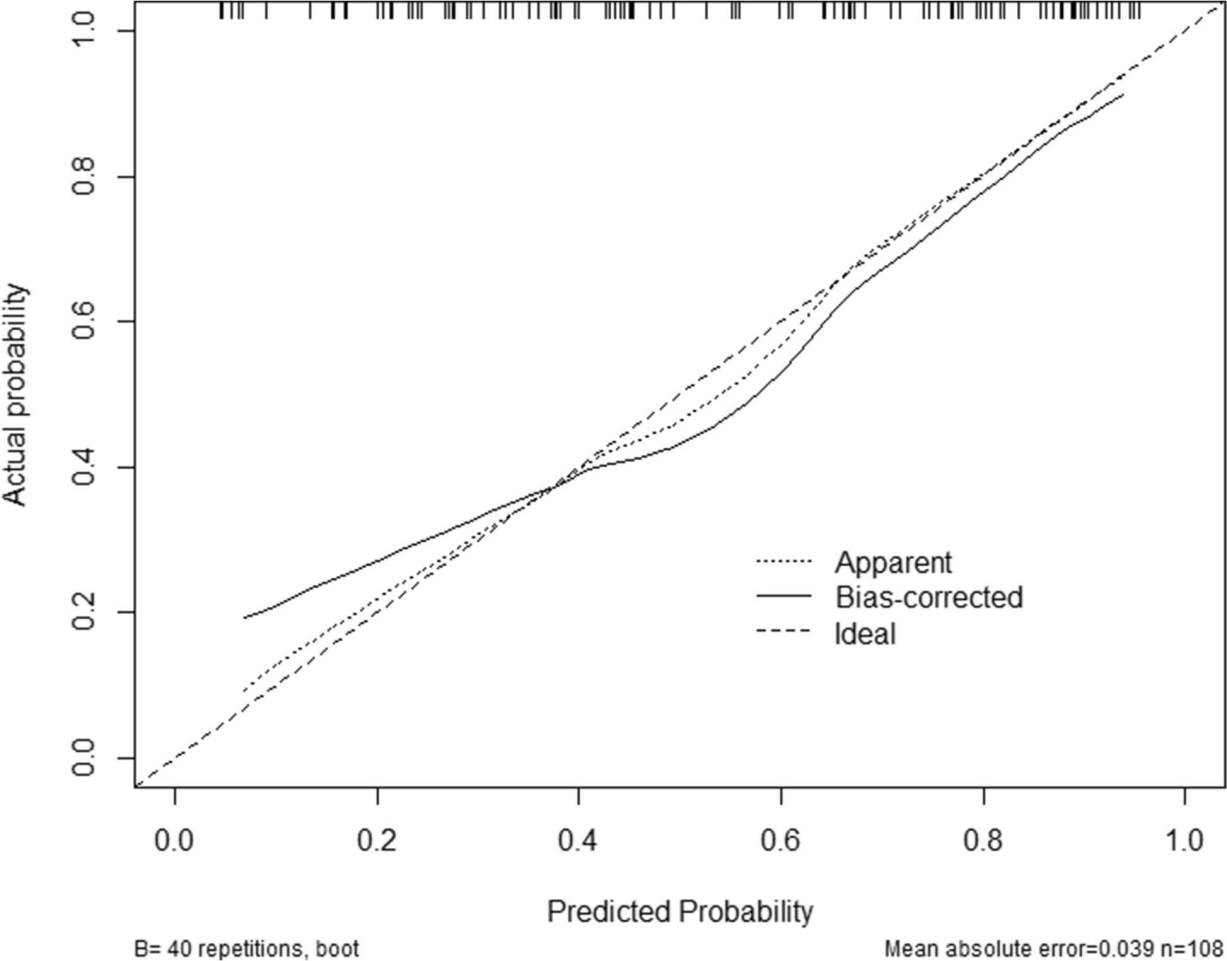
Calibration curves of the nomogram model for predicting PTG in children with malignancies. B ¼ 1000 repetitions, boot mean absolute error ¼ 0.039, n ¼ 108. The x-axis shows the predicted occurrence of PTG, and the y-axis shows the observed occurrence of PTG. The dotted and solid lines indicate the observed (apparent) nomogram performance before and after bootstrapping

The nomogram that could predict the occurrence of PTG was created using the factors of age, diagnosis, educational level of the caregiver, level of self-efficacy and coping style of the child (Fig. 1). The area under the ROC curve was 0.837 (Fig. 2). The model with the highest Youden index defined PTG as 0.566, with a sensitivity of 83.1% and a specificity of 73.5%. The 108 children with malignancies were used as a verification group to validate the model. The C-indexes of internal and external validation were 0.837 (95%CI:0786~0.886) and 0.813 (95%CI:0732~0.894), respectively, indicating a high discrimination of the nomogram. The calibration plots also showed high coherence between the predicted occurrence of PTG and the actual observations (Fig. 3), which indicated good calibration of the model. And, according to Fig. 1, we can see that the specific scale on the line segment corresponding to each of the five variables represents the range of values available for that variable, while the length of the line segment reflects the size of the variable’s contribution to the ending event.

## Discussions

### PTG in Chinese children aged 8–18 years with malignancies was low

The results of this study showed that the percentage of children with malignancies who had PTG was 26.0% (65/250), and the total score on the PTG scale was (43.42 ± 17.43), which was significantly lower than those in previous studies (41.13% [30] and 53.30 ± 20.22 [23]). The reason for this difference may be that children aged 8–18 years are in a period of psychological defiance, they are sensitive, and emotionally unstable, and experiencing a traumatic event such as a tumor can intensify their negative emotions, which be detrimental to PTG production [31]. Repeated hospitalization, invasive operations and complications can cause traumatic stress, as can overprotective caregivers and a closed social environment [9]. Additionally, influenced by traditional Chinese culture, caregivers are not sufficiently aware of the rehabilitation care of children with malignancies and can overemphasize children’s illness [32] and use approaches such as control and restrict activities to overprotect them [33]. This hinders the development of their early independent consciousness and socially adaptive behavior, which is not conducive to PTG. Therefore, it is important to clarify the core factors of PTG in Chinese children aged 8–18 years with malignancies and construct a prediction model to enhance their coping and adaptation abilities.

### PTG in Chinese children aged 8–18 years with malignancies was influenced by multiple factors

#### Age

The study found that older age was a protective predictor of PTG in children aged 8–18 years with malignancies. This finding fits with the report by Turner JK et al. [34]. This might be because older children have greater access to information and a deeper understanding of illness and tend to develop mature cognitive resources and self-awareness, which leads to more positive reflections on trauma [35]. Moreover, the effect of age on PTG in Chinese children with malignancies was found to be no different from that in other countries by comparison [10, 34]. We suggest that nursing staff can provide younger children with more simple methods of psychological suggestion such as sand tray games, music therapy, and drawing to promote PTG.

#### Diagnosis

This study shows that solid tumors can promote PTG production in children with malignant tumors. This is contrary to the findings of Wang Jingjing et al. [30]. It may be related to the children with osteosarcoma in this study had an onset at age 12 or older. There is a strong capacity for posttraumatic cognitive reconstruction for older children to engage in positive psychological adjustment, which are conducive to PTG [34]. But, a Korean study [36] showed that children with solid tumors were less prone to PTG because they were treated for a shorter period of time than children with hematological tumors. Therefore, follow-up studies should further explore the specific mechanism.

#### Educational level of the caregiver

As in previous studies, the educational level of the caregiver was also closely related to PTG in children with malignancies [22]. The reasons why children with highly-educated caregivers have better PTG might be as follows: They have a better understanding of disease and treatment, are more psychologically resilient, which can provide more informational resources and support to the children, facilitating the children’s psychological and behavioral adjustments [37]. Additionally, China is a developing country, and the education level of the caregivers in this study was predominantly junior or senior high school, generally inferior to that in developed countries. Therefore, we recommend that nursing staff take measures such as positive parenting courses [38] and parent management training to raise caregiver’s awareness to provide more social support.

#### Coping style of the child

We found that children with negative coping styles had a lower probability of PTG. Morris et al. [39] also showed that more adaptive coping styles were associated with higher PTG. This may be the positive coping style can help children view traumatic experiences such as tumors optimistically and reduce the impact of negative emotions that can cause PTG. Unlike developed countries, such as Europe, where more educated parents place a high value on independent thinking and self-direction of their children40, this study found that Chinese children with malignancies cope mostly with refusal and resistance [40]. Caregivers are overly constrained and caring, which is not conducive to the positive development of the child’s self-response. We recommend that nursing staff instruct caregivers to help the children with appropriate exercise and socialization and allow them to handle tasks within their abilities.

#### Level of self-efficacy

Based on Fig. 1, we found that self-efficacy had the greatest effect on PTG. This finding is also consistent with previously reported findings that increased self-efficacy can improve PTG in cancer patients [41]. It is possible that self-efficacy allows affected children to increase their sense of control, helps them view traumatic experiences as opportunities for growth. In this study, 93.2% (233/250) of the children had low or medium levels of self-efficacy, significantly lower than Japanese [42] and Korean [43]. It is obvious that the self-efficacy level of Chinese children aged 8–18 years with malignancies needs to be improved. We recommend that nursing staff invite recovered children to share their experiences, help children in treatment learn self-confidence and communication skills through regular sharing sessions or health seminars, and provide appropriate psychological support to improve PTG.

### The nomogram model constructed could well predict the probability of PTG in Chinese children aged 8–18 years with malignancies

We included five predictor variables related to PTG in our logistic regression analysis. The high C-index and consistent calibration curve indicate that the model has good discrimination and calibration. Distinguishing between high and low PTG levels based on the optimal threshold of 0.566 will not only allow early screening for adverse psychological problems but also help health care professionals promote PTG with intervention strategies. Additionally, we found that the level of self-efficacy had a greater impact on PTG in Chinese children with malignancies aged 8–18 years than other factors in this study. This indicates that the follow-up intervention should focus on the regulation of the children’s intrinsic self-mechanisms.

This study had some limitations. First, only a single tertiary children’s hospital was included, with a single source of data. Second, PTG is a psychological change process, but due to sample size and model limitations, some influencing factors, such as network information found in clinical observations, peer support, and support received from health care providers during treatment, may be missed. And dynamic longitudinal observations were not conducted in this study, providing a weaker explanation of causality. Future studies can use a longitudinal design to further explore the relationship between different treatment phases and PTG in children with malignancies.

## Conclusions

Our study suggests that age, diagnosis, coping style of the child, educational level of the caregiver and level of self-efficacy are independent predictors of PTG in children aged 8–18 years with malignancies, and provide a reference basis for clinical nursing to conduct psychological interventions for children with malignancies and, to a certain extent, help them recover after trauma.

## Data Availability

Research data are not shared. (The datasets used and/or analysed during the current study are available from the corresponding author on reasonable request).
